# 5,6,7,8-Tetra­hydro­quinolin-8-one

**DOI:** 10.1107/S1600536811016175

**Published:** 2011-05-07

**Authors:** Teodozja M. Lipińska, Zbigniew Karczmarzyk, Waldemar Wysocki, Ewa Gruba, Andrzej Fruziński

**Affiliations:** a Department of Chemistry, University of Podlasie, ul. 3 Maja 54, 08-110 Siedlce, Poland; b Department of General and Ecological Chemistry, Technical University, ul. Żeromskiego 115, 90-924 Łódź, Poland

## Abstract

In the quinoline fused-ring system of the title compound, C_9_H_9_NO, the pyridine ring is planar to within 0.011 (3) Å, while the partially saturated cyclo­hexene ring adopts a sofa conformation with an asymmetry parameter Δ*C*_s_(C6) = 1.5 (4)°. There are no classical hydrogen bonds in the crystal structure. Mol­ecules form mol­ecular layers parallel to (100) with a distance between the layers of *a*/2 = 3.468 Å.

## Related literature

The title compound is an inter­mediate for the synthesis of polyheterocycles giving photoluminescence (Kelly & Lebedev, 2002 ▶) and a key substrate to synthesis of its 8-amino substituted derivatives with pharmacological activity (*e.g.* Gudmundsson *et al.*, 2009) ▶. For our ongoing study on the synthesis and structure of condensed pyridine and quinoline derivatives, see: Lipińska (2005 ▶); Karczmarzyk *et al.* (2010 ▶). For the synthesis, see: Kelly & Lebedev (2002 ▶). For a related structure, see: OXHYQU (Cygler *et al.*, 1981 ▶). For structure inter­pretation tools, see: Bruno *et al.* (2002 ▶); Spek (2009 ▶). For a description of the Cambridge Structural Database, see: Allen (2002 ▶). For bond-length data, see: Allen *et al.* (1987 ▶). For asymmetry parameters, see: Duax & Norton (1975 ▶).
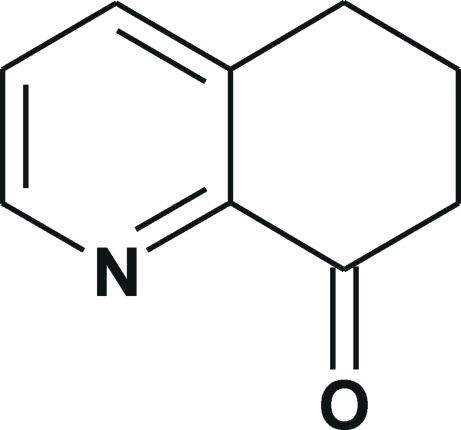


## Experimental

### 

#### Crystal data

C_9_H_9_NO
*M* *_r_* = 147.17
Orthorhombic, 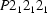

*a* = 6.9393 (2) Å
*b* = 8.0885 (3) Å
*c* = 13.4710 (4) Å
*V* = 756.11 (4) Å^3^
*Z* = 4
Cu *K*α radiation
μ = 0.68 mm^−1^
*T* = 293 K
0.60 × 0.16 × 0.15 mm

#### Data collection

Bruker SMART APEXII CCD diffractometer
Absorption correction: multi-scan (*SADABS*; Bruker, 2005 ▶) *T*_min_ = 0.878, *T*_max_ = 1.000
5358 measured reflections
761 independent reflections
734 reflections with *I* > 2σ(*I*)
*R*_int_ = 0.022

#### Refinement

*R*[*F* ^2^ > 2σ(*F* ^2^)] = 0.050
*wR*(*F* ^2^) = 0.153
*S* = 1.14
761 reflections
100 parameters
H-atom parameters constrained
Δρ_max_ = 0.28 e Å^−3^
Δρ_min_ = −0.18 e Å^−3^

### 

Data collection: *APEX2* (Bruker, 2005 ▶); cell refinement: *SAINT* (Bruker, 2005 ▶); data reduction: *SAINT*; program(s) used to solve structure: *SHELXS97* (Sheldrick, 2008 ▶); program(s) used to refine structure: *SHELXL97* (Sheldrick, 2008 ▶); molecular graphics: *ORTEP-3 for Windows* (Farrugia, 1997 ▶); software used to prepare material for publication: *SHELXL97* and *WinGX* (Farrugia, 1999 ▶).

## Supplementary Material

Crystal structure: contains datablocks I, global. DOI: 10.1107/S1600536811016175/jh2281sup1.cif

Structure factors: contains datablocks I. DOI: 10.1107/S1600536811016175/jh2281Isup2.hkl

Supplementary material file. DOI: 10.1107/S1600536811016175/jh2281Isup3.cml

Additional supplementary materials:  crystallographic information; 3D view; checkCIF report

## Figures and Tables

**Fig. 1.**
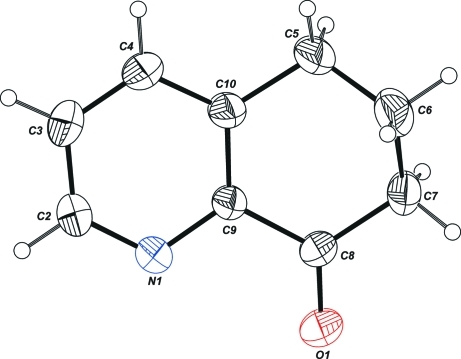
The molecular structure of (I), with atom labels and 30% probability displacement ellipsoids for non-H atoms.

**Fig. 2.**
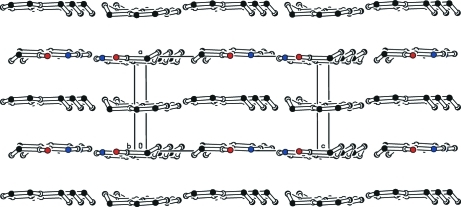
A view of the molecular packing in (I).

## supplementary crystallographic information

### Comment

6,7-Didro-5*H*-quinolin-8-one, (I), is an important intermediate for the synthesis of polyheterocycles giving photoluminescence (Kelly & Lebedev, 2002) and a key substrate to synthesis of its 8-amino substituted derivatives with pharmacological activity (*e.g.* Gudmundsson *et al.*, 2009). As a part of our ongoing study on the synthesis and structure of condensed pyridine and quinoline derivatives (Lipińska, 2005; Karczmarzyk *et al.*, 2010) we report herein the X-ray structure of the title compound. This compound is well-known on organic chemistry but its crystal structure is not current in the Cambridge Structural Database (November 2010 Release; Allen, 2002; Bruno *et al.*, 2002).

The bond lengths and angles for (I) are within expected ranges (Allen *et al.*, 1987) and are comparable to the corresponding values observed in related structure of 8-oxo-2-phenyl-5,6,7,8-tetrahydroquinoline (OXHYQU; CSD, November 2010 Release). In the two-ring fused system the aromatic pyridine ring is planar within 0.011 (3) Å, while the partially saturated cyclohexene ring adopts a sofa conformation with asymmetry parameter Δ*C_S_*(C6) = 1.5 (4)° (Duax & Norton, 1975).

There are no classical hydrogen bonds in the crystal structure of (I). The nearly planar molecules form molecular layers parallel to (100) crystallographic plane (Fig. 2) imposing in the unit cell the pseudo-mirror plane passing through N, O, C(*sp*^2^) and C5(*sp*^3^) atoms (higher pseudosymmetry *Pnma* space group). The distance between neighbouring planes of *a*/2 = 3.468 Å is comparable to a van der Waals distance of about 3.5 Å for the *π*-*π* interacting aromatic skeletons of pyridine rings.

### Experimental

The titled compound was obtained by ozonolysis of 8-benzylidene-5,6,7,8-tetrahydroquinoline according to the method described by Kelly & Lebedev (2002). Crystals suitable for X-ray diffraction analysis were grown by slow evaporation of a ethyl acetate/hexane (1:1) solution.

### Refinement

The H atoms were positioned geometrically and treated as riding on their C atoms, with C—H distances of 0.93 (aromatic) and 0.97 Å (CH_2_), and were refined with *U*_ĩso_(H) values of 1.5*U*_eq_(C). The Flack parameter originally was refined to 0.4 (6), which is essentially indeterminate. For this reason, the Friedel equivalents were merged using MERG4 in *SHELXL97* (Sheldrick, 2008) and the absolute structure was arbitrarily assigned. The *PLATON* symmetry check (Spek, 2009) reveals the presence of pseudosymmetry in the structure suggesting the higher symmetry space group *Pnma* in the unit cell with the cell constants of *a*' = *b*, *b*' = *a* and *c*' = *c* and origin shifted to:-0.2500, 0.2577, 0.0000. This pseudosymmetry forces the molecule to locate on the crystallographic mirror plane passing through N, O, all C(*sp*^2^) and C5(*sp*^3^) atoms and C6 atom to be disordered over two positions above and below the mirror plane. The attempt to refine the structure in the *Pnma* space group resulted in a more disordered model with high *R* and *wR* values of 0.199 and 0.527, respectively.

### Crystal data

**Table:**

C_9_H_9_NO	*D*_x_ = 1.293 Mg m^−^^3^
*M**_r_* = 147.17	Melting point = 369–371 K
Orthorhombic, *P*2_1_2_1_2_1_	Cu *K*α radiation, λ = 1.54178 Å
Hall symbol: P 2ac 2ab	Cell parameters from 40 reflections
*a* = 6.9393 (2) Å	θ = 7.2–34.8°
*b* = 8.0885 (3) Å	µ = 0.68 mm^−^^1^
*c* = 13.4710 (4) Å	*T* = 293 K
*V* = 756.11 (4) Å^3^	Needle, colourless
*Z* = 4	0.60 × 0.16 × 0.15 mm
*F*(000) = 312	

### Data collection

**Table:**

Bruker SMART APEXII CCD diffractometer	761 independent reflections
Radiation source: fine-focus sealed tube	734 reflections with *I* > 2σ(*I*)
graphite	*R*_int_ = 0.022
ω scans	θ_max_ = 65.0°, θ_min_ = 6.4°
Absorption correction: multi-scan (*SADABS*; Bruker, 2005)	*h* = −8→8
*T*_min_ = 0.878, *T*_max_ = 1.000	*k* = −9→7
5358 measured reflections	*l* = −15→15

### Refinement

**Table:**

Refinement on *F*^2^	Primary atom site location: structure-invariant direct methods
Least-squares matrix: full	Secondary atom site location: difference Fourier map
*R*[*F*^2^ > 2σ(*F*^2^)] = 0.050	Hydrogen site location: inferred from neighbouring sites
*wR*(*F*^2^) = 0.153	H-atom parameters constrained
*S* = 1.14	*w* = 1/[σ^2^(*F*_o_^2^) + (0.1138*P*)^2^ + 0.0433*P*] where *P* = (*F*_o_^2^ + 2*F*_c_^2^)/3
761 reflections	(Δ/σ)_max_ < 0.001
100 parameters	Δρ_max_ = 0.28 e Å^−^^3^
0 restraints	Δρ_min_ = −0.18 e Å^−^^3^

### Special details

**Table:**

Geometry. All esds (except the esd in the dihedral angle between two l.s. planes) are estimated using the full covariance matrix. The cell esds are taken into account individually in the estimation of esds in distances, angles and torsion angles; correlations between esds in cell parameters are only used when they are defined by crystal symmetry. An approximate (isotropic) treatment of cell esds is used for estimating esds involving l.s. planes.
Refinement. Refinement of F^2^ against ALL reflections. The weighted R-factor wR and goodness of fit S are based on F^2^, conventional R-factors R are based on F, with F set to zero for negative F^2^. The threshold expression of F^2^ > 2sigma(F^2^) is used only for calculating R-factors(gt) etc. and is not relevant to the choice of reflections for refinement. R-factors based on F^2^ are statistically about twice as large as those based on F, and R- factors based on ALL data will be even larger.

### Fractional atomic coordinates and isotropic or equivalent isotropic displacement parameters (Å^2^)

**Table:**

	*x*	*y*	*z*	*U*_iso_*/*U*_eq_	
O1	0.0077 (5)	0.3562 (2)	0.54598 (13)	0.0827 (8)	
N1	0.0144 (4)	0.0574 (3)	0.64019 (14)	0.0617 (6)	
C2	0.0232 (4)	−0.0911 (4)	0.68161 (18)	0.0673 (7)	
H2	0.0116	−0.0986	0.7502	0.101*	
C3	0.0486 (5)	−0.2350 (4)	0.6282 (2)	0.0763 (9)	
H3	0.0575	−0.3364	0.6604	0.114*	
C4	0.0606 (6)	−0.2257 (3)	0.5270 (2)	0.0784 (10)	
H4	0.0776	−0.3212	0.4896	0.118*	
C5	0.0569 (7)	−0.0575 (4)	0.36824 (19)	0.0850 (12)	
H51	0.1908	−0.0533	0.3478	0.128*	
H52	−0.0010	−0.1544	0.3381	0.128*	
C6	−0.0431 (6)	0.0917 (4)	0.3321 (2)	0.0899 (11)	
H61	−0.1807	0.0782	0.3419	0.135*	
H62	−0.0203	0.1031	0.2613	0.135*	
C7	0.0218 (5)	0.2459 (3)	0.38350 (19)	0.0685 (8)	
H71	−0.0596	0.3370	0.3626	0.103*	
H72	0.1526	0.2708	0.3630	0.103*	
C8	0.0157 (4)	0.2338 (3)	0.49507 (19)	0.0547 (6)	
C9	0.0250 (3)	0.0646 (3)	0.54022 (17)	0.0498 (6)	
C10	0.0472 (4)	−0.0740 (3)	0.48064 (18)	0.0593 (7)	

### Atomic displacement parameters (Å^2^)

**Table:**

	*U*^11^	*U*^22^	*U*^33^	*U*^12^	*U*^13^	*U*^23^
O1	0.1330 (18)	0.0423 (10)	0.0727 (12)	−0.0004 (12)	0.0010 (14)	−0.0078 (7)
N1	0.0823 (14)	0.0571 (12)	0.0455 (10)	0.0067 (12)	0.0018 (10)	−0.0021 (8)
C2	0.0834 (17)	0.0693 (15)	0.0491 (12)	0.0051 (16)	0.0046 (12)	0.0103 (11)
C3	0.099 (2)	0.0553 (16)	0.0748 (16)	0.0030 (15)	0.0054 (16)	0.0198 (12)
C4	0.121 (3)	0.0434 (15)	0.0710 (17)	0.0049 (16)	0.0102 (17)	−0.0019 (11)
C5	0.146 (3)	0.0603 (17)	0.0493 (14)	−0.0020 (19)	0.0052 (16)	−0.0100 (11)
C6	0.141 (3)	0.080 (2)	0.0489 (13)	−0.011 (2)	−0.0132 (16)	0.0019 (13)
C7	0.0942 (18)	0.0564 (14)	0.0549 (13)	−0.0025 (14)	−0.0030 (14)	0.0134 (11)
C8	0.0668 (13)	0.0410 (12)	0.0563 (12)	−0.0016 (11)	0.0007 (12)	−0.0020 (9)
C9	0.0616 (12)	0.0445 (12)	0.0434 (10)	−0.0013 (11)	0.0012 (10)	−0.0015 (8)
C10	0.0805 (16)	0.0462 (13)	0.0512 (13)	−0.0051 (13)	0.0066 (11)	−0.0042 (10)

### Geometric parameters (Å, °)

**Table:**

O1—C8	1.205 (3)	C5—H51	0.9700
N1—C2	1.326 (3)	C5—H52	0.9700
N1—C9	1.350 (3)	C6—C7	1.497 (4)
C2—C3	1.380 (4)	C6—H61	0.9700
C2—H2	0.9300	C6—H62	0.9700
C3—C4	1.368 (4)	C7—C8	1.507 (3)
C3—H3	0.9300	C7—H71	0.9700
C4—C10	1.380 (4)	C7—H72	0.9700
C4—H4	0.9300	C8—C9	1.499 (3)
C5—C6	1.475 (5)	C9—C10	1.387 (3)
C5—C10	1.521 (3)		
			
C2—N1—C9	117.2 (2)	C5—C6—H62	109.0
N1—C2—C3	123.4 (2)	C7—C6—H62	109.0
N1—C2—H2	118.3	H61—C6—H62	107.8
C3—C2—H2	118.3	C6—C7—C8	113.5 (2)
C4—C3—C2	118.7 (2)	C6—C7—H71	108.9
C4—C3—H3	120.6	C8—C7—H71	108.9
C2—C3—H3	120.6	C6—C7—H72	108.9
C3—C4—C10	119.7 (2)	C8—C7—H72	108.9
C3—C4—H4	120.1	H71—C7—H72	107.7
C10—C4—H4	120.1	O1—C8—C9	121.4 (2)
C6—C5—C10	112.3 (3)	O1—C8—C7	121.0 (2)
C6—C5—H51	109.1	C9—C8—C7	117.57 (19)
C10—C5—H51	109.1	N1—C9—C10	123.3 (2)
C6—C5—H52	109.1	N1—C9—C8	116.21 (19)
C10—C5—H52	109.1	C10—C9—C8	120.5 (2)
H51—C5—H52	107.9	C4—C10—C9	117.7 (2)
C5—C6—C7	112.8 (3)	C4—C10—C5	121.7 (2)
C5—C6—H61	109.0	C9—C10—C5	120.7 (2)
C7—C6—H61	109.0		
			
C9—N1—C2—C3	2.2 (4)	O1—C8—C9—C10	−175.6 (3)
N1—C2—C3—C4	−1.8 (5)	C7—C8—C9—C10	2.9 (4)
C2—C3—C4—C10	0.1 (6)	C3—C4—C10—C9	1.0 (5)
C10—C5—C6—C7	52.3 (4)	C3—C4—C10—C5	−179.1 (4)
C5—C6—C7—C8	−51.6 (4)	N1—C9—C10—C4	−0.5 (4)
C6—C7—C8—O1	−158.0 (3)	C8—C9—C10—C4	178.1 (3)
C6—C7—C8—C9	23.5 (4)	N1—C9—C10—C5	179.6 (3)
C2—N1—C9—C10	−1.0 (4)	C8—C9—C10—C5	−1.8 (4)
C2—N1—C9—C8	−179.7 (2)	C6—C5—C10—C4	154.3 (3)
O1—C8—C9—N1	3.1 (4)	C6—C5—C10—C9	−25.9 (5)
C7—C8—C9—N1	−178.4 (3)		
